# Supplementary material for: Protective and vulnerability personality traits associated with PTSD diagnosis after preterm delivery
Source: PLoS One. 2024 Aug 12;19(8):e0308498. doi: 10.1371/journal.pone.0308498 (PMC11318917; doi:10.1371/journal.pone.0308498)
Supplement: S1 Data — (PDF) [file pone.0308498.s001.pdf]

Participant	Group	dob_mother	level_study	Nb_pregnancies	Nb_birth	antdt_psy1	antdt_psy2	care	Birth_date
FA01	control	avr.-85	15	1	1	depression		0	07/09/2018
HG03	control	avr.-85	17	2	1			0	03/10/2018
LL06	control	mars-99	10	1	1	depression		1	20/05/2018
JK08	control	janv.-00	9	1	1			0	23/09/2018
PM11	control	juil.-94	14	1	1			0	08/08/2018
VK12	control	mars-91	14	1	1			0	11/05/2018
SF13	control	janv.-80	10	5	3			0	01/06/2018
LC14	control	avr.-86	11	1	1	depression		1	17/08/2018
DL15	control	mai-97	14	1	1			0	23/12/2018
FR17	control	mai-94	15	1	1			0	07/08/2018
ME18	control	déc.-84	14	1	1			0	25/10/2018
FO20	control	juil.-86	15	1	1			0	09/01/2018
PR21	control	févr.-97	10	4	2			0	06/11/2018
FC22	control	août-87	14	2	1			0	08/04/2018
IP23	control	juil.-80	11	6	6	depression		0	31/12/2018
LS24	control	nov.-92	15	1	2			0	30/12/2018
MM26	control	févr.-83	13	1	1			0	30/03/2018
ZS27	control	déc.-87	11	3	3			0	05/03/2018
CA28	control	déc.-96	11	3	1			0	07/01/2018
AP30	control	mai-81	18	4	1			0	30/12/2018
DM31	control	oct.-91	15	1	2	Burnout		0	01/12/2018
AS32	control	août-81	15	1	1			0	17/04/2018
ZA33	control	févr.-82	10	6	8			0	31/01/2018
AB34	control	déc.-89	12	2	2			0	23/01/2018
DS35	control	mars-86	14	1	1			1	19/02/2018
TK36	control	janv.-81	15	2	2			0	16/01/2018
CG39	control	Jan-93	11	7	4			0	29/11/2018
GE41	control	Aug-90	15	2	2			0	10/11/2018
GM43	control	May-99	11	1	1			0	24/12/2017
WJ49	control	Sep-89	15	1	1			1	08/01/2019
SN48	control	Nov-85	17	6	3			0	21/01/2019
CS47	control	Mar-83	16	1	2			1	21/01/2019
BA02	PTSD	juin-96	14	1	1	parents_separation		1	02/01/2018

Participant	Group	dob_mother	level_study	Nb_pregnancies	Nb_birth	antdt_psy1	antdt_psy2	care	Birth_date
PZ04	PTSD	juil.-89	14	1	1	depression		1	08/09/2018
LD05	PTSD	sept.-81	10	6	3			0	02/05/2018
DJ07	PTSD	juil.-96	16	1	1	depression	Anorexia	1	11/05/2018
PC09	PTSD	juin-84	17	2	2			0	22/04/2018
GM10	PTSD	avr.-83	16	2	2			0	04/06/2018
FK16	PTSD	avr.-83	11	5	2	depression		1	15/11/2018
LG25	PTSD	avr.-90	11	2	1			0	24/02/2018
BC29	PTSD	janv.-82	11	3	2	depression		1	23/02/2018
MD37	PTSD	févr.-91	12	3	2	Post partum depression		1	28/12/2017
FS38	PTSD	févr.-83	12	2	2			0	26/11/2018
MA40	PTSD	Jul-00	9	2	2			0	29/10/2018
LS42	PTSD	Oct-95	9	1	1			0	07/12/2018
AJ44	PTSD	aout-95	12	1	1			0	11/12/2018
CL50	PTSD	Mar-94	9	1	1			0	06/01/2019
MK45	PTSD	Nov-97	10	3	2	anxiety		1	01/01/2019
TS51	control	Feb-83	CAP	2	2			0	20/03/2019
MB52	control	Feb-87	17	1	2			0	19/03/2019
FA53	control	Jan-91	15	2	3			0	15/03/2019

Participant	Date_theoritical	Gestational_age	baby_weight	APGAR1	APGAR3	APGAR5	Crane_size	IUGR	Nb_babies
FA01	03/11/2018	229	1800	4	5		28,5	1	1
HG03	29/12/2018	202	770	9	10	10	24	1	1
LL06	24/08/2018	191	970	3	6	10	25	0	1
JK08	08/12/2018	210	1600	9	10	8	28	0	1
PM11	01/12/2018	174	740	2			21	0	1
VK12	11/08/2018	189	1035	5	7	8	23	0	1
SF13	26/08/2018	196	1230	8	9	9	26	0	0
LC14	21/10/2018	222	1270	5	7	8	25,08	1	1
DL15	03/03/2019	217	1380	6	8	8	25,08	0	1
FR17	29/11/2018	173	635	2			20	0	2
ME18	01/01/2019	219	910	8	10	10	25	1	1
FO20	08/04/2018	199	1084	3	7	8	25,08	0	2
PR21	29/01/2019	203	1175	9	7	9	26	0	1
FC22	10/06/2018	224	1198	4	9	10	25,08	0	1
IP23	17/03/2019	210	1060	8	10	10	25,50	0	2
LS24	03/03/2019	224	830	6	8	9	29	1	2
MM26	22/07/2018	173	705	0	3		22,00	0	1
ZS27	03/06/2018	196	1020	7	9	9	25	0	1
CA28	30/03/2018	205	1190	4	5	8	27	0	1
AP30	29/03/2019	197	760	4	7	8	25	1	1
DM31	22/03/2019	175	700	7	8	10	23	0	2
AS32	13/07/2018	196	770	4	8	10	22	1	1
ZA33	15/04/2018	213	1620	5	8	10	29	0	2
AB34	25/04/2018	195	1039	8	9	10	35	0	2
DS35	06/06/2018	180	640	5	8	9	21	1	1
TK36	05/04/2018	209	1360	5	8	9	28	0	2
CG39	04/02/2019	217	1125	4	7	8	25	1	1
GE41	09/02/2019	196	790	8	9	9	23,5	1	1
GM43	04/04/2018	182	900	6			22,5	0	1
WJ49	24/03/2019	210	1160					0	1
SN48	01/05/2019	182	650					1	1
CS47	10/04/2019	207	1165					0	2
BA02	21/04/2018	175	820	1			23	0	1

Participant	Date_theoritical	Gestational_age	baby_weight	APGAR1	APGAR3	APGAR5	Crane_size	IUGR	Nb_babies
PZ04	30/12/2018	176	780	6	10	10	24	0	1
LD05	02/08/2018	189	1180	8	9	10	25,08	0	1
DJ07	25/07/2018	212	950	9	10	10	26	1	1
PC09	31/07/2018	182	845	6			24	0	2
GM10	14/08/2018	212	1265	5	6	8	27	0	1
FK16	24/02/2019	188	625	4	2	intubé	22,00	1	1
LG25	06/05/2019	216	1250	6	3	9	25,08	0	1
BC29	27/04/2018	221	845	9	10	10	27	1	2
MD37	08/03/2018	215	1140	5	8	10	26	1	1
FS38	18/03/2019	176	670	6	10	10	22	0	2
MA40	19/01/2019	208	1430	8	9	9	24,5	0	1
LS42	27/02/2019	203	1360	3	6	8	25,5	0	1
AJ44	13/03/2019	195	1055	8	9	10	25	0	1
CL50	23/03/2019	211	1120					0	1
MK45	20/03/2019	209	1560	9	10,00	10,00	27,5	0	2
TS51	08/07/2019	25+2	760				22	0	1
MB52	23/05/2019	31+4	1040					1	2
FA53	10/06/2019	28	1150					0	2

Participant	Prem_birth_risk	Hospitalization	Delivery_mode	Anesthesia	midwife_debrief	father_support	Env_support	PPQ
FA01	0	0	VB	Aucune	0	1	1	5
HG03	1	1	C-section	Rachi	0	1	0	2
LL06	0	0	VB	Péri	0	1	1	4
JK08	1	1	C-section	Générale	0	1	1	1
PM11	0	0	VB	Péri	1	1	1	4
VK12	1	1	VB	Péri	0	1	0	5
SF13	0	1	VB	Péri	0	0	1	4
LC14	0	0	C-section	Rachi	0	1	0	4
DL15	1	1	C-section	Rachi	0	1	1	5
FR17	1	1	VB	Péri	0	1	1	3
ME18	0	1	c-section	Rachi	0	1	1	4
FO20	1	1	c-section	Rachi	0	1	1	0
PR21	1	1	c-section	Péri	0	1	1	3
FC22	0	0	c-section	Générale	0	1	1	1
IP23	0	0	c-section	Générale	0	1	1	5
LS24	1	1	c-section	Rachi	0	1	1	2
MM26	0	0	VB	Péri	0	1	1	4
ZS27	1	1	VB	Aucune	0	1	0	3
CA28	0	0	VB	Péri	0	1	1	2
AP30	0	1	C-section	Rachi	0	1	1	0
DM31	1	1	VB	Péridurale	0	1	1	4
AS32	0	0	C-section	Péridurale	0	1	1	3
ZA33	0	1	C-section	Rachi	0	0	1	1
AB34	0	0	VB	Péri	0	1	1	1
DS35	1	1	C-section	Rachi	0	1	1	5
TK36	1	1	VB	Morphine	0	1	1	0
CG39	1	1	C-section	Rachi	0	1	1	0
GE41	0	0	C-section	Rachi	0	1	1	3
GM43	0	0	VB	Aucune	0	1	1	4
WJ49	0	0	C-section	Générale	1	1	1	3
SN48	1	0	C-section	Rachi	1	1	1	2
CS47	1	1	VB	Péri	1	&	&	0
BA02	0	0	VB	Péri	0	1	1	7

Participant	Prem_birth_risk	Hospitalization	Delivery_mode	Anesthesia	midwife_debrief	father_support	Env_support	PPQ
PZ04	0	0	C-section	Générale	0	1	1	9
LD05	1	0	VB	Aucune	1	1	1	6
DJ07	0	1	C-section	Rachi	0	0	0	6
PC09	1	1	VB	Péri	1	1	1	8
GM10	0	1	C-section	Rachi	0	0	1	9
FK16	1	1	C-section	Générale	0	1	0	8
LG25	0	0	C-section	Générale	0	1	1	6
BC29	1	1	C-section	Rachi	0	1	0	7
MD37	1	1	C-section	Générale	0	1	1	6
FS38	0	0	VB	Péri	0	0	1	6
MA40	1	1	VB	Péri	0	1	0	8
LS42	0	0	VB	Péri	0	1	0	6
AJ44	0	1	C-section	Rachi	1	1	1	6
CL50	0	0	C-section	Générale	0	1	1	10
MK45	1	1	C-section	Rachi	0	1	1	7
TS51	0	0	VB	Péri	1	1	1	0
MB52	1	1	C-section	Rachi	0	1	1	2
FA53	1	1	VB	Péri	0	1	0	5

Participant	CAPS_A	CAPS_B	CAPS_C	CAPS_D	CAPS_E	CAPS_Total	CAPS_Nb_Sx	CAPS_F	CAPS_G	Cotes
FA01										
HG03										
LL06										
JK08										
PM11										
VK12										
SF13										
LC14										
DL15										
FR17										
ME18										
FO20										
PR21										
FC22										
IP23										
LS24										
MM26										
ZS27										
CA28										
AP30										
DM31										
AS32										
ZA33										
AB34										
DS35										
TK36										
CG39										
GE41										
GM43										
WJ49										
SN48										
CS47										
BA02	1	8	3	8	6	25	9	1	3	

Participant	CAPS_A	CAPS_B	CAPS_C	CAPS_D	CAPS_E	CAPS_Total	CAPS_Nb_Sx	CAPS_F	CAPS_G	Cotes
PZ04	1	12	6	7	5	30	12	1	5	
LD05	1	10.5	1	12.5	8	32	10	1	5	
DJ07	1	5	0	4	12	21	8	1		
PC09	1	7	2	11	0	20	7	1	2.5	
GM10	1	7	3	10	4	24	10	1	2	
FK16	1	5	3	11	7	26	10	1	3	
LG25	1	5	4	11	5	25	9	1	2	
BC29	1	8.5	6	4.5	8	27	10	1		
MD37	1	7	3,5	16	7	34,5	10	1		
FS38	1	8,5	3	14	7,5	33	11	1		
MA40	1	6	0	13	14	33	10	1	2	
LS42	1	3	0	11	9	23	9	1	2	
AJ44	1	3	0	7	6	16	6	1	3	
CL50										
MK45										
TS51										
MB52										
FA53										

Participant	CAPS_Dissoc	CAPS_Score	O	C	E	A	N		
FA01			23	42	25	22	33		
HG03			22	27	31	28	14		
LL06			31	44	35	36	14		
JK08			23	37	26	46	2		
PM11			16	35	37	30	31		
VK12			20	35	30	34	30		
SF13			14	37	36	37	21		
LC14			19	28	29	35	35		
DL15			23	36	29	36	28		
FR17			25	38	22	39	9		
ME18			38	41	35	33	25		
FO20			18	35	36	27	13		
PR21			18	30	20	23	22		
FC22			24	46	44	47	1		
IP23			31	38	29	40	39		
LS24			13	43	39	37	6		
MM26			17	33	26	36	20		
ZS27			25	38	32	24	31		
CA28			22	38	36	27	21		
AP30			23	38	34	37	13		
DM31			21	33	24	36	16		
AS32			18	43	34	37	10		
ZA33			22	36	30	32	17		
AB34			23	30	32	31	16		
DS35			17	41	29	38	14		
TK36			28	37	39	34	10		
CG39			28	34	29	30	21		
GE41			25	37	24	31	24		
GM43			23	47	29	36	17		
WJ49			24	39	28	33	26		
SN48			26	46	39	27	8		
CS47			21	33	31	40	24		
BA02	0	1,25	23	40	33	39	23		

Participant	CAPS_Dissoc	CAPS_Score	O	C	E	A	N		
PZ04	0	1,5	33	29	30	35	16		
LD05	0	1,6	20	33	28	38	31		
DJ07	0	1,05	40	25	35	31	28		
PC09	0	1	30	19	32	29	31		
GM10		1,3	11	36	28	41	13		
FK16	0	1,3	35	34	32	27	14		
LG25		1,25	16	20	9	36	35		
BC29		1,35	32	33	22	23	27		
MD37		1,73	10	45	22	31	6		
FS38		1,65	18	43	28	39	31		
MA40	0	1,7	19	37	20	17	18		
LS42	0	1,15	13	32	20	27	28		
AJ44	0	0,8	24	45	35	27	28		
CL50			27	41	31	28	37		
MK45			24	41	24	43	17		
TS51			24	24	24	33	27		
MB52			13	43	31	34	12		
FA53			35	41	24	28	21		

