# Supplementary material for: Protective and vulnerability personality traits associated with PTSD diagnosis after preterm delivery
Source: PLoS One. 2024 Aug 12;19(8):e0308498. doi: 10.1371/journal.pone.0308498 (PMC11318917; doi:10.1371/journal.pone.0308498)
Supplement: Attachment — Submitted filename: Response to Reviewers-1.docx [file pone.0308498.s002.docx]

Response to Reviewers

Dear Reviewer,

Thank you for your recommendation on our paper. We really appreciate the time and consideration you put into our work. Here are our answers to the questions and remarks you raised:

1. *Please ensure that your manuscript meets PLOS ONE's style requirements, including those for file naming*

We have edited our document to meet the PLOS ONE’s style requirements in terms of font and titles.

2. *Please amend your current ethics statement to address the consent form:*

We have added a few information addressing this question in our paper in further details. Yes, participants received a written document that they signed. This question was addressed in the ethical part, we added it in the participant paragraph as well following your concerns.

*3. In your Data Availability statement, you have not specified where the minimal data set underlying the results described in your manuscript can be found. PLOS defines a study's minimal data set as the underlying data used to reach the conclusions drawn in the manuscript and any additional data required to replicate the reported study findings in their entirety. All PLOS journals require that the minimal data set be made fully available.*

We see no restriction in making our data public. It was a mistake on our end not to share them. All relevant data are within the manuscript and its Supporting Information files.

**Comments to the author:**

We added tables and all our data to strengthen our paper as per recommended. We added the multiple regression table, the Cronbach’s alphas of our scales and all data used to lead to our results.

Again, thank you very much for your feedback and recommendation. We hope we were able to improve our paper to meet your Journal’s requirements and contribute to new findings in our field.
