# Supplementary material for: Protective and vulnerability personality traits associated with PTSD diagnosis after preterm delivery
Source: PLoS One. 2024 Aug 12;19(8):e0308498. doi: 10.1371/journal.pone.0308498 (PMC11318917; doi:10.1371/journal.pone.0308498)
Supplement: Attachment — Submitted filename: Response to Reviewers.docx [file pone.0308498.s003.docx]

Response to Reviewers

Dear Reviewer,

Thank you for your recommendation on our paper. We really appreciate the time and consideration you put into our work. Here are our answers to the questions and remarks you raised. Each reviewer will be addressed individually.

We considered the comments of reviewer 2 and edited our paper as per his or her suggestion when it was possible. We modified the title, added some citations, made sure we were consistent in writing our decimals and rewrote some part in order to improve our paper. Although we are sorry our study does not meet your expectations, we appreciate your recommendation to improve it.

**Reviewer 3:**

Thank you very much for your comment and the time you spent reviewing our paper. We are glad our paper met your expectations and agree on the interest and importance this field of research has.

**Reviewer 4:**

Thank you for your time and the comments you made in order to improve our paper. Here are our responses to each of your point:

TITLE

1. *Please include that your study assessed personality traits, perhaps “Protective and vulnerability personality traits associated with PTSD diagnosis after preterm delivery.”*

We modified the title as suggested to better reflect our study.

INTRODUCTION
*1. Line 69 – I’m not sure that cerebral immaturity is a reason for stress in parents of preterm infants, all babies are cerebrally immature. Do you perhaps mean the potential for neurodevelopmental adverse outcomes in the preterm period? Please explain this thought more.*

Maybe we were not clear enough. Although we do not think cerebral immaturity is the cause of stress in parents of preterm infants, research show that those babies are less likely to respond back to social cues which is highly important in the development of the bond.

*2. Line 84-86 – stress has also been shown to cause premature activation of the hypothalamic pituitary axis, and may be a risk factor for preterm birth, I think this should be addressed/discussed.*

This is a very interesting point that we have considered. Although we are aware of studies such as the study of Julia Seng (among others) which indeed found a correlation between prenatal stress in mothers and preterm birth, we decided not to add this perspective in our paper. Mothers who gave birth prematurely already show intense feelings of guilt and as clinicians, we did not want to add to it. Moreover, although it would have been very interesting to measure the stress in mothers before they gave birth (at T0) to have a comparison level, it would have been logistically very complicated. It would imply meeting every pregnant woman not knowing if they are actually going to give birth prematurely and be included in the study.

We have added this limit to our study in the discussion part. Thank you for this feedback.

*3. Line 97-98, you note that ”Research on this subject estimates that it occurs in 25% to 33% of mothers after a preterm birth.” What were the risk factors for the development of PTSD in mothers who experienced preterm birth. Please add this to the discussion here, as it makes the reader interested to find out more from your study.*

We did! You are right. Thank you for your comment.

*4. Line 132, you note that ”Certain personality traits, notably extraversion and 133 neuroticism, are strongly correlated with the emergence of anxiety disorders.” However, there is no citation for this statement. Please include one as well as additional rationale for choosing only these 2 of the 5 personality traits to include in this study.*

We tested all 5 personality traits in our study but only the two we had expected (extraversion and neuroticism) showed a correlation. Our hypotheses only focused on these two as they are often found in other studies as correlated with our interest here (depression, anxiety, etc). Citations 24 to 26 are studies that show correlations between those two traits and the development of pathologies like depression or anxiety and level of stress. I added a line in the discussion to clarify that we did assess all five dimensions in our study.

MATERIALS AND METHODS
*1. Line 154 – you note that mothers whose babies died were not included in the study. Please discuss your rationale for this decision. Also I think you should discuss later how this may have affected your results. By excluding participants whose babies died, I think you are skewing the results. People whose babies died are more likely to experience PTSD from the event.*

This is indeed a decision we made for several reasons:

- Women whose infants died do not stay at the hospital and we would have been very difficult for us to include them for the study
- For ethical reasons and because we are clinicians, it seems to us that taking part in a research shortly after experience child loss would not have been beneficial for women
- Finally, comparing women whose baby died with women whose baby did not could be another study by itself. One is experiencing symbolic mourning (of the ideal baby) while the other is experiencing real grief (real as in “the reality”).

I added it in the discussion section to help the reader understand our choice.

2. Please include the validity, sensitivity/specificity and positive/negative predictive value of the PPQ and NEO-FFI surveys.

The internal consistency is high. Our Cronbach’s alphas are consistent with those of the original test (ranging from .68 for Agreeableness to .89 for Neuroticism). A two-week retest reliability was made for the NEO-FFI in 2001 (Robins & al., 2001) and showed scores from .86 to .90. The NEO inventories scales have been widely used to measure personality.

As for the PPQ, the internal consistency is good with Cronbach’s alphas of .73 and .74 (internal consistency of the two factors in the PPQ). The retest reliability done one month apart is .92. Its validity has been measured by comparing the PPQ with IES (Impact of Event Scale) obtaining a .78 correlation between the two instruments. (Pierrehumbert & al., 2004).

3. Was a sample size calculated for this study? Please include this information.

There was no sample size calculated for this study as we conducted a primary and prospective survey in 2017 which already showed promising results and the same correlations. Hence our wish to continue the study and to include as many participants as we could that following year.

DISCUSSION
*1. Please include a discussion of how the results may have been impacted by the exclusion of mothers whose babies died.*

We added the rationale behind it in the discussion as suggested.

3. *I think you should also discuss how your results were likely influenced by the inclusion of mothers of preterm infants born at < 33 weeks (morse severe than infants born between 34 and 36 weeks (late preterm)*

You are right. We made the choice of focusing on mothers of preterm infants born at < 33 weeks because they stay at the hospital longer and can be more easily included in research. I will specify it throughout the paper as saying “preterm birth” is not precise enough as we only focused on severe prematurity. Thank you.
