# Supplementary material for: Protective and vulnerability personality traits associated with PTSD diagnosis after preterm delivery
Source: PLoS One. 2024 Aug 12;19(8):e0308498. doi: 10.1371/journal.pone.0308498 (PMC11318917; doi:10.1371/journal.pone.0308498)
Supplement: Attachment — Submitted filename: Response to Reviewers-4.docx [file pone.0308498.s004.docx]

Response to Reviewers

Dear Reviewers,

Thank you for the time spent on reviewing our paper. We edited our manuscript based on your suggestions.

*Reviewer 5:*

- Table 4. We added the Cohen d value and confidence intervals as suggested. The Cohen d value for this analysis was .83 and confidence intervals of 95% limits were [0,32 ; 3,92]. Everything was added in the paper.
- Table 5. We also added the F of the multiple regression which is 2,64 (p=.04) as well as the unstandardized betas.

Reviewer 6:

- I deleted the question as suggested and added a few lines to introduce the next paragraph.
- There are no prior study studying the link between personality traits and PTSD. As explained in our paper, past research studied external factors (baby weight, etc) and found no correlation. We then decided to focus on internal factors such as personality traits. We later explained how personality traits can be correlated to such pathologies mentioning several studies on that matter. I am not sure what or how to change.
- I added a paragraph to further develop our ideas on the topic. Thank you!

Reviewer 7:

- Thank you for your feedback, however if I may correct one thing: all five traits were explored but only two were significant. We mentioned that we did test all five of them (line 226).
- The environmental factors that you mentioned are very interesting and we did test some of them (social support or obstetric history for example but they were not significant, line 231).
- I understand your idea for a follow-up study on the effects of PSTD which I find very interesting. However, there are already some research of the topic and we decided to focus on the vulnerability and protections factors to PTSD, not PTSD itself for our research. Our main goal was to better identify women at risk and prevent the risk for PTSD.
- Your suggestions are very appreciated, I added them on our discussion and will keep them in mind for future research!
